# (*E*)-Methyl *N*′-[1-(4-hydroxy­phen­yl)ethyl­idene]hydrazinecarboxyl­ate

**DOI:** 10.1107/S1600536808024227

**Published:** 2008-08-06

**Authors:** Lu-Ping Lv, Jian-Wu Xie, Wen-Bo Yu, Wei-Wei Li, Xian-Chao Hu

**Affiliations:** a Department of Chemical Engineering, Hangzhou Vocational and Technical College, Hangzhou 310018, People’s Republic of China; b Research Center of Analysis and Measurement, Zhejiang University of Technology, Hangzhou 310014, People’s Republic of China

## Abstract

The title compound, C_10_H_12_N_2_O_3_, adopts a *trans* configuration with respect to the C=N bond. The dihedral angle between the benzene ring and the hydrazine carboxylic acid plane is 8.29 (7)°. Mol­ecules are linked into a three-dimensional network by N—H⋯O, O—H⋯O, O—H⋯N hydrogen bonds and C—H⋯π inter­actions.

## Related literature

For general background, see: Parashar *et al.* (1988 ▶); Hadjoudis *et al.* (1987 ▶); Borg *et al.* (1999 ▶). For related structures, see: Shang *et al.* (2007 ▶).
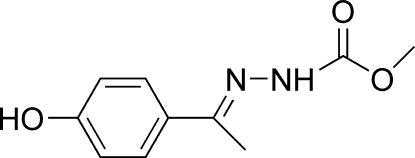


## Experimental

### 

#### Crystal data

C_10_H_12_N_2_O_3_
*M* *_r_* = 208.22
Orthorhombic, 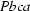

*a* = 11.2532 (18) Å
*b* = 10.4310 (17) Å
*c* = 17.226 (3) Å
*V* = 2022.1 (6) Å^3^
*Z* = 8
Mo *K*α radiation
μ = 0.10 mm^−1^
*T* = 273 (2) K
0.31 × 0.27 × 0.25 mm

#### Data collection

Bruker SMART CCD area-detector diffractometer
Absorption correction: multi-scan (*SADABS*; Bruker, 2002 ▶) *T*_min_ = 0.972, *T*_max_ = 0.978
12287 measured reflections
1794 independent reflections
1626 reflections with *I* > 2σ(*I*)
*R*_int_ = 0.016

#### Refinement

*R*[*F* ^2^ > 2σ(*F* ^2^)] = 0.033
*wR*(*F* ^2^) = 0.098
*S* = 1.09
1794 reflections
140 parameters
H-atom parameters constrained
Δρ_max_ = 0.17 e Å^−3^
Δρ_min_ = −0.15 e Å^−3^

### 

Data collection: *SMART* (Bruker, 2002 ▶); cell refinement: *SAINT* (Bruker, 2002 ▶); data reduction: *SAINT*; program(s) used to solve structure: *SHELXS97* (Sheldrick, 2008 ▶); program(s) used to refine structure: *SHELXL97* (Sheldrick, 2008 ▶); molecular graphics: *SHELXTL* (Sheldrick, 2008 ▶); software used to prepare material for publication: *SHELXTL*.

## Supplementary Material

Crystal structure: contains datablocks I, global. DOI: 10.1107/S1600536808024227/bg2200sup1.cif

Structure factors: contains datablocks I. DOI: 10.1107/S1600536808024227/bg2200Isup2.hkl

Additional supplementary materials:  crystallographic information; 3D view; checkCIF report

## Figures and Tables

**Table 1:** Hydrogen-bond geometry (Å, °)

*D*—H⋯*A*	*D*—H	H⋯*A*	*D*⋯*A*	*D*—H⋯*A*
O1—H1⋯O21^i^	0.82	2.61	3.0523 (14)	116
O1—H1⋯N1^i^	0.82	2.03	2.8464 (14)	174
N2—H2*A*⋯O2^ii^	0.86	2.44	3.2777 (16)	164
C10—H10*B*⋯*Cg*1^iii^	0.96	2.90	3.7169 (19)	144

Symmetry codes: (i) 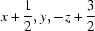; (ii) 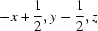; (iii) 
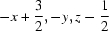
. *Cg*1 is the centroid of the C1–C6 ring.

**Fig. 1.**
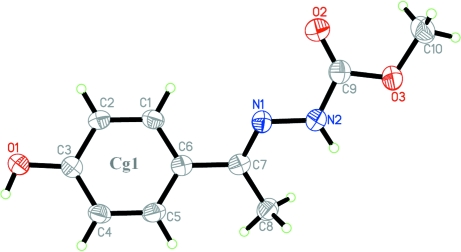
Molecular structure of (I), showing 50% probability displacement ellipsoids and the atomic numbering.

## supplementary crystallographic information

### Comment

Benzaldehydehydrazone derivatives have received considerable attentions for a long time due to their pharmacological activity (Parashar *et al.*, 1988) and their photochromic properties(Hadjoudis *et al.*, 1987). Meanwhile, it is an important intermediate of 1,3,4-oxadiazoles, which have been reported to be versatile compounds with interesting properties (Borg *et al.*, 1999). As a further investigation of this type of derivatives, the crystal structure of the title compound, C_10_H_12_N_2_O_3_(Fig.1), is described here.

The title molecule (Fig.1) adopts a *trans* configuration with respect to the C═N bond. The N1/N2/O2/O3/C9/C10 plane of the hydrazine carboxylic acid methyl ester group is slightly twisted away from the attached ring, to which it subtends a dihedral angle of 8.29 (7)°. Bond lengths and angles agree with those observed for methyl *N*'-[(*E*)-4-methoxybenzylidene] hydrazinecarboxylate (Shang *et al.*, 2007).

The molecules are linked into a three-dimensional network by N–H···O, O–H···O, O–H···N hydrogen bonds and C–H···π interactions. (Table 1).

### Experimental

4-Hydroxy-acetophenone (1.36 g, 0.01 mol) and methyl hydrazinecarboxylate (0.90 g, 0.01 mol) were dissolved in stirred methanol (25 ml) and left for 4 h at room temperature. The resulting solid was filtered off and recrystallized from ethanol to give the title compound in 80% yield. Crystals suitable for X-ray analysis were obtained by slow evaporation of a ethanol solution at room temperature (m.p. 483–485 K).

### Refinement

H atoms were included in the riding model approximation with N—H = 0.86Å and O—H =0.82 Å. C-bound H atoms were positioned geometrically (C—H = 0.93 or 0.96 Å) and refined using a riding model, with *U*_iso_(H) = 1.2–1.5*U*_eq_(C).

### Crystal data

**Table:**

C_10_H_12_N_2_O_3_	*F*_000_ = 880
*M**_r_* = 208.22	*D*_x_ = 1.368 Mg m^−^^3^
Orthorhombic, *P**b**c**a*	Mo *K*α radiation λ = 0.71073 Å
Hall symbol: -P 2ac 2ab	Cell parameters from 1794 reflections
*a* = 11.2532 (18) Å	θ = 2.4–25.0º
*b* = 10.4310 (17) Å	µ = 0.10 mm^−^^1^
*c* = 17.226 (3) Å	*T* = 273 (2) K
*V* = 2022.1 (6) Å^3^	Block, colourless
*Z* = 8	0.31 × 0.27 × 0.25 mm

### Data collection

**Table:**

Bruker SMART CCD area-detector diffractometer	1794 independent reflections
Radiation source: fine-focus sealed tube	1626 reflections with *I* > 2σ(*I*)
Monochromator: graphite	*R*_int_ = 0.016
*T* = 273(2) K	θ_max_ = 25.1º
φ and ω scans	θ_min_ = 2.4º
Absorption correction: multi-scan(SADABS; Bruker, 2002)	*h* = −13→10
*T*_min_ = 0.972, *T*_max_ = 0.978	*k* = −12→12
12287 measured reflections	*l* = −20→20

### Refinement

**Table:**

Refinement on *F*^2^	Hydrogen site location: inferred from neighbouring sites
Least-squares matrix: full	H-atom parameters constrained
*R*[*F*^2^ > 2σ(*F*^2^)] = 0.033	*w* = 1/[σ^2^(*F*_o_^2^) + (0.0493*P*)^2^ + 0.6007*P*] where *P* = (*F*_o_^2^ + 2*F*_c_^2^)/3
*wR*(*F*^2^) = 0.098	(Δ/σ)_max_ = 0.001
*S* = 1.09	Δρ_max_ = 0.17 e Å^−^^3^
1794 reflections	Δρ_min_ = −0.15 e Å^−^^3^
140 parameters	Extinction correction: SHELXL97 (Sheldrick, 2008), Fc^*^=kFc[1+0.001xFc^2^λ^3^/sin(2θ)]^-1/4^
Primary atom site location: structure-invariant direct methods	Extinction coefficient: 0.0032 (8)
Secondary atom site location: difference Fourier map	

### Special details

**Table:**

Geometry. All e.s.d.'s (except the e.s.d. in the dihedral angle between two l.s. planes) are estimated using the full covariance matrix. The cell e.s.d.'s are taken into account individually in the estimation of e.s.d.'s in distances, angles and torsion angles; correlations between e.s.d.'s in cell parameters are only used when they are defined by crystal symmetry. An approximate (isotropic) treatment of cell e.s.d.'s is used for estimating e.s.d.'s involving l.s. planes.
Refinement. Refinement of *F*^2^ against ALL reflections. The weighted *R*-factor *wR* and goodness of fit *S* are based on *F*^2^, conventional *R*-factors *R* are based on *F*, with *F* set to zero for negative *F*^2^. The threshold expression of *F*^2^ > σ(*F*^2^) is used only for calculating *R*-factors(gt) *etc*. and is not relevant to the choice of reflections for refinement. *R*-factors based on *F*^2^ are statistically about twice as large as those based on *F*, and *R*- factors based on ALL data will be even larger.

### Fractional atomic coordinates and isotropic or equivalent isotropic displacement parameters (Å^2^)

**Table:**

	*x*	*y*	*z*	*U*_iso_*/*U*_eq_	
C1	0.54458 (12)	0.13035 (13)	0.82724 (8)	0.0330 (3)	
H3	0.4976	0.1806	0.8596	0.040*	
C7	0.47041 (11)	−0.06505 (12)	0.89717 (7)	0.0287 (3)	
C5	0.61590 (11)	−0.07467 (13)	0.78629 (8)	0.0329 (3)	
H5	0.6177	−0.1634	0.7913	0.039*	
C9	0.22859 (11)	0.01890 (13)	1.01445 (7)	0.0332 (3)	
C3	0.68423 (11)	0.11609 (13)	0.72183 (7)	0.0294 (3)	
C2	0.61307 (12)	0.18868 (13)	0.77120 (8)	0.0339 (3)	
H2	0.6118	0.2775	0.7663	0.041*	
C6	0.54416 (11)	−0.00311 (12)	0.83650 (7)	0.0283 (3)	
C4	0.68424 (12)	−0.01681 (13)	0.72942 (7)	0.0328 (3)	
H4	0.7302	−0.0667	0.6963	0.039*	
C8	0.50358 (13)	−0.19457 (13)	0.92764 (8)	0.0366 (3)	
H8A	0.5024	−0.1932	0.9834	0.055*	
H8B	0.5819	−0.2166	0.9100	0.055*	
H8C	0.4477	−0.2571	0.9091	0.055*	
N2	0.30927 (11)	−0.05735 (10)	0.97785 (7)	0.0348 (3)	
H2A	0.3164	−0.1372	0.9895	0.042*	
N1	0.37946 (9)	−0.00106 (10)	0.92106 (6)	0.0299 (3)	
O1	0.74919 (9)	0.18049 (9)	0.66825 (5)	0.0381 (3)	
H1	0.7828	0.1291	0.6397	0.057*	
O3	0.16440 (10)	−0.05274 (10)	1.06350 (6)	0.0471 (3)	
O2	0.21727 (9)	0.13304 (9)	1.00534 (6)	0.0433 (3)	
C10	0.07785 (16)	0.01597 (17)	1.10905 (10)	0.0540 (5)	
H10A	0.1108	0.0965	1.1253	0.081*	
H10B	0.0571	−0.0339	1.1539	0.081*	
H10C	0.0081	0.0310	1.0783	0.081*	

### Atomic displacement parameters (Å^2^)

**Table:**

	*U*^11^	*U*^22^	*U*^33^	*U*^12^	*U*^13^	*U*^23^
C1	0.0343 (7)	0.0289 (7)	0.0359 (7)	0.0019 (6)	0.0053 (6)	−0.0057 (5)
C7	0.0261 (6)	0.0289 (7)	0.0309 (7)	−0.0016 (5)	−0.0063 (5)	−0.0016 (5)
C5	0.0315 (7)	0.0245 (7)	0.0426 (8)	0.0020 (5)	−0.0003 (6)	−0.0014 (5)
C9	0.0302 (7)	0.0359 (8)	0.0334 (7)	−0.0036 (6)	−0.0026 (5)	−0.0005 (6)
C3	0.0268 (6)	0.0316 (7)	0.0298 (6)	−0.0015 (5)	−0.0012 (5)	−0.0024 (5)
C2	0.0390 (8)	0.0238 (6)	0.0390 (7)	0.0006 (5)	0.0041 (6)	−0.0025 (5)
C6	0.0249 (6)	0.0286 (7)	0.0315 (7)	−0.0005 (5)	−0.0036 (5)	−0.0013 (5)
C4	0.0309 (7)	0.0300 (7)	0.0374 (7)	0.0043 (6)	0.0039 (5)	−0.0051 (5)
C8	0.0345 (7)	0.0321 (7)	0.0432 (8)	−0.0003 (6)	0.0006 (6)	0.0048 (6)
N2	0.0366 (7)	0.0293 (6)	0.0383 (6)	−0.0002 (5)	0.0056 (5)	0.0042 (5)
N1	0.0290 (6)	0.0319 (6)	0.0289 (6)	−0.0010 (5)	0.0005 (4)	0.0029 (4)
O1	0.0434 (6)	0.0314 (5)	0.0396 (5)	0.0003 (4)	0.0132 (4)	−0.0021 (4)
O3	0.0500 (7)	0.0373 (6)	0.0541 (6)	−0.0066 (5)	0.0232 (5)	−0.0039 (5)
O2	0.0437 (6)	0.0358 (6)	0.0505 (6)	0.0056 (5)	0.0053 (5)	0.0056 (5)
C10	0.0557 (10)	0.0507 (10)	0.0558 (10)	−0.0061 (8)	0.0227 (8)	−0.0134 (8)

### Geometric parameters (Å, °)

**Table:**

C1—C2	1.3770 (19)	C3—C4	1.3924 (19)
C1—C6	1.4012 (19)	C2—H2	0.9300
C1—H3	0.9300	C4—H4	0.9300
C7—N1	1.2894 (17)	C8—H8A	0.9600
C7—C6	1.4827 (18)	C8—H8B	0.9600
C7—C8	1.4967 (18)	C8—H8C	0.9600
C5—C4	1.3840 (19)	N2—N1	1.3877 (15)
C5—C6	1.3990 (18)	N2—H2A	0.8600
C5—H5	0.9300	O1—H1	0.8200
C9—O2	1.2076 (17)	O3—C10	1.4416 (18)
C9—O3	1.3394 (16)	C10—H10A	0.9600
C9—N2	1.3618 (18)	C10—H10B	0.9600
C3—O1	1.3556 (16)	C10—H10C	0.9600
C3—C2	1.3920 (18)		
			
C2—C1—C6	121.38 (12)	C5—C4—C3	120.04 (12)
C2—C1—H3	119.3	C5—C4—H4	120.0
C6—C1—H3	119.3	C3—C4—H4	120.0
N1—C7—C6	116.34 (11)	C7—C8—H8A	109.5
N1—C7—C8	123.60 (12)	C7—C8—H8B	109.5
C6—C7—C8	120.07 (11)	H8A—C8—H8B	109.5
C4—C5—C6	121.71 (12)	C7—C8—H8C	109.5
C4—C5—H5	119.1	H8A—C8—H8C	109.5
C6—C5—H5	119.1	H8B—C8—H8C	109.5
O2—C9—O3	125.11 (12)	C9—N2—N1	117.31 (11)
O2—C9—N2	125.88 (12)	C9—N2—H2A	121.3
O3—C9—N2	109.01 (12)	N1—N2—H2A	121.3
O1—C3—C2	117.17 (12)	C7—N1—N2	117.25 (11)
O1—C3—C4	123.86 (11)	C3—O1—H1	109.5
C2—C3—C4	118.96 (12)	C9—O3—C10	115.49 (12)
C1—C2—C3	120.66 (12)	O3—C10—H10A	109.5
C1—C2—H2	119.7	O3—C10—H10B	109.5
C3—C2—H2	119.7	H10A—C10—H10B	109.5
C5—C6—C1	117.25 (12)	O3—C10—H10C	109.5
C5—C6—C7	121.75 (12)	H10A—C10—H10C	109.5
C1—C6—C7	121.01 (11)	H10B—C10—H10C	109.5
			
C6—C1—C2—C3	0.0 (2)	C6—C5—C4—C3	1.0 (2)
O1—C3—C2—C1	−179.91 (12)	O1—C3—C4—C5	179.43 (12)
C4—C3—C2—C1	0.66 (19)	C2—C3—C4—C5	−1.17 (19)
C4—C5—C6—C1	−0.33 (18)	O2—C9—N2—N1	−4.8 (2)
C4—C5—C6—C7	179.66 (12)	O3—C9—N2—N1	176.23 (11)
C2—C1—C6—C5	−0.20 (19)	C6—C7—N1—N2	−179.99 (10)
C2—C1—C6—C7	179.81 (12)	C8—C7—N1—N2	−0.36 (18)
N1—C7—C6—C5	−156.37 (12)	C9—N2—N1—C7	166.88 (11)
C8—C7—C6—C5	23.98 (18)	O2—C9—O3—C10	−1.4 (2)
N1—C7—C6—C1	23.61 (17)	N2—C9—O3—C10	177.62 (12)
C8—C7—C6—C1	−156.03 (12)		

### Hydrogen-bond geometry (Å, °)

**Table:**

*D*—H···*A*	*D*—H	H···*A*	*D*···*A*	*D*—H···*A*
O1—H1···O2^i^	0.82	2.61	3.0523 (14)	116
O1—H1···N1^i^	0.82	2.03	2.8464 (14)	174
N2—H2A···O2^ii^	0.86	2.44	3.2777 (16)	164
C10—H10B···Cg1^iii^	0.96	2.90	3.7169 (19)	144

Symmetry codes: (i) *x*+1/2, *y*, −*z*+3/2; (ii) −*x*+1/2, *y*−1/2, *z*; (iii) −*x*+3/2, −*y*, *z*−1/2.
